# The impact of resilience on anxiety and depression among grass-roots civil servants in China

**DOI:** 10.1186/s12889-021-10710-2

**Published:** 2021-04-13

**Authors:** Huiling Song, Mengjie Zhang, Yanjie Wang, Liying Yang, Yanyu Wang, Yan Li

**Affiliations:** 1 Department of Children, Adolescents and Women Health, College of Public Health, Zhengzhou University, 100 Kexue Road, Zhengzhou, 450000 Henan China; 2 The First Affiliated Hospital and College of Clinical Medicine of Henan University of Science and Technology, 24 Jinghua Road, Luoyang, 4571003 Henan China; 3 School of Physical Education (Main Campus), Zhengzhou University, Zhengzhou, Henan China

**Keywords:** Grass-roots civil servants, Stress, Depression, Anxiety, Resilience

## Abstract

**Background:**

The grass-roots civil servants are the final implementers and executors of a series of government policies and the fundamental force for social stability and harmonious development. However, the mental health problems of grass-roots civil servants have not got full attention. This study aimed to assess the impact of resilience on anxiety and depression among grass-roots civil servants in China.

**Method:**

From Oct to Dec 2019, 302 civil servants completed a series of questionnaires. The Civil Servants Stress Scale (CSSS) was used to assess the stress of civil servants. The Self-rating Depression Scale (SDS) and the Self-rating Anxiety Scale (SAS) were used to evaluate the depression and anxiety of participants, respectively. The resilience of civil servants evaluates by the Connor-Davidson Resilience Scale (CD-RSCI). We conducted the moderating and mediating analysis on the impact of resilience on depression and anxiety in grass-roots civil servants.

**Results:**

There were significant differences in gender, education, position, relationship with coworkers, physical exercise, and monthly income for stress in grass-roots civil servants (*P* < 0.05). Resilience can negatively regulate the stress of grass-roots civil servants, and an effective mediator and moderator in the relationship between stress and anxiety and depression and the mediating effect ratios of 7.77 and 22.79%.

**Conclusion:**

Resilience has moderating and mediating effects on the relationship between stress and depression, and anxiety. The negative effects of stress on depression and anxiety of grass-roots civil servants can be buffered by resilience as a dynamic moderator directly and indirectly. These findings contribute to society and government better understand the mental health status of grass-roots civil servants and provide references and guidance for the formulation of corresponding management and prevention measures.

## Background

A civil servant is a person who performs public responsibilities, whose salary and benefits are provided by the state national public finance and brought in the state administrative management system [1]. Civil servants play an important role in the modernization of China as the manager and executor of state affairs. However, to achieve the objective of building a moderately affluent society in an all-around way in China, implementing a job accountability system and the reform of rank promotion led to more rigorous assessment standards and the rare chance of promotion in civil servants [2]. Therefore, the occupational pressure and social pressure that civil servants are facing are constantly increasing [3]. Multiple studies have shown that the special work pressure of civil servants is related to psychological health, such as depression, anxiety, and burnout of civil servants [4–6]. The grass-roots civil servants are the ultimate implementers and executors of a series of government policies and the fundamental force for social stability and harmonious development [7]. In terms of rank, this part of the group is the personnel at the bottom of the rank sequence, and the post settings are mostly clerks and few cadres. At the grass-roots civil servants, cadres are generally responsible for the assignment and acceptance of tasks, while clerks are responsible for the specific implementation of tasks. The salaries of grass-roots civil servants are generally stable but relatively low, and lack of leadership position makes it difficult for them to be promoted. All these may lead to increasing occupational stress of the grass-roots civil servants. Many kinds of research have shown that career stress could induce physical illness and some psychological problems [8–10]. For grass-roots civil servants, stress threatens their health and the work efficiency of the government and social stability. But the mental health problems of grass-roots civil servants in China have not got full attention. According to a recent study, the suicide rate of Chinese civil servants is higher than that of any other occupation in the country and higher than that of civil servants in foreign [11]. This reminded us that paying close attention to the mental health of grass-roots civil servants is essential.

Past research had shown that long-term exposure to stress hormones throughout the lifespan could affect brain structures involved in recognition and mental health [12]. These affected brain structures could lead to the differences in response to stress, cognition, and memory of the brain [12]. Besides, chronic stress is associated with mental disorders and chronic diseases [13, 14] psychological problems [15]. When stress reaches a certain level and can be not handled properly, it could lead to anxiety, depression, neurasthenia, burnout, and other clinical symptoms [16]. Between 1990 to 2013, the number of people all over the world suffering from depression or anxiety or comorbidities increased by nearly 50%, from 416 million to 615 million, according to statistics [17]. Although it is not clear that the pathogenesis of depression and anxiety, some studies have found a strong correlation between stress and the occurrence of depression and anxiety [18, 19]. Depression and anxiety are often accompanied by pressure, such as great academic stress leads more than half of medical students to have depression, anxiety, and so on [16, 20–24].

The concept of resilience is a process of dynamic change [25]. Resilience generally refers to the ability to overcome the stress or adverse situation or to resist the environmental risk [26]. A broader definition of resilience refers to the ability of a dynamic system to bear or recover from major challenges that threaten its stability, survival, or development. It can be said that resilience is the ability of human beings to adapt in the face of sadness, adversity, and constant and significant stress in life [27]. Simply put, resilience can be regarded as a protective factor within individuals, which is of great significance to relieve pressure and promote mental health [28, 29]. At present, there are mainly three models about resilience, which are: compensation model, protection model, and challenge model [30, 31]. The direct effects were usually used to test the compensatory model of resilience, and interaction in multiple regressions was used to test the protective model [32]. In this study, we tested the compensatory and protective models of resilience. Studies have shown that resilience mediated the relationship between stress, depression, and anxiety and had a protective effect on adolescents and pregnant women [27, 32]. Hence, we supposed that resilience could potentially improve coping strategies to avoid the negative effects of stress on mental health, such as depression and anxiety among civil servants. Many studies provided results either from qualitative research or limited to stress with physical health [33, 34]. However, there were few studies on the relationship between mental health problems and stress in grass-roots civil servants. It is reported that resilience affect not only the subjective well-being of civil servants and moderate the relationship between stress and subjective well-being [35]. However, there is still a lack of research on the resilience of grass-roots civil servants and its effect on anxiety and depression. It is not clear whether its protective effect is direct or indirect. For the first time, this study investigated the psychological status of grass-roots civil servants, analyzed the current situation of stress, depression, and anxiety faced by the grass-roots civil servants. This study also analyzed the impact of the resilience of grass-roots civil servants on stress, depression, and anxiety to fills in the blank of previous research.

**Fig. 1:**
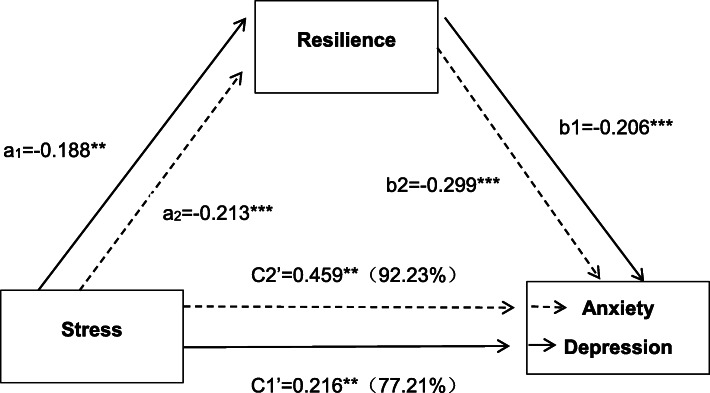
Mediating effects of resilience in the relationship between stress (short for stress) and depression (solid line), anxiety (dash line) in civil servants (*N* = 302). Note: *P < 05, **P < < 01,***P < 001

So, we made the hypotheses shown in Fig. 1: (1) Resilience can mediate the relationship between stress and depression and anxiety. (2) Resilience can moderate the relationship between stress and depression and anxiety.

## Method

### Participants

From October to December 2019, we randomly selected one sample from 8 counties in Xinyang, Henan province, and carried out cluster sampling for 12 county-level units in Shangcheng County, which selected. We counted 350 grass-roots civil servants on the job in 12 units during the investigation period, among which 330 participants cooperated with the survey, with a response rate of 94.3%. We used the field survey; all the respondents were asked to fill in the questionnaire, and the questionnaire was collected on the spot. The investigators checked the questionnaire on spot to make sure it was complete. Finally, after excluding invalid questionnaires such as regular answers, a total of 302 questionnaires were selected with an effective rate of 91.5%. The Science Ethics Committee approved the research of Zhengzhou University. Written informed consent was obtained from each participant before starting any investigation related to the study.

### Demographic data

Demographic characteristics included gender, age, an only child family (the respondent is the only child in the family), marital status, degree of education, seniority (years of work of the respondents), position, relationship with colleagues, physical exercise, and monthly income.

### Civil servants stress scale (CSSS)

The CSSS is a 38-item self-report scale divided into 6 dimensions: management and development, life relationships, work relationships, health and responsibility, economic stress, and workload. The CSSS is rated on a 10-point scale, from 1 (no stress) to 10(extreme stress). The scores range from 0 to 380, and the higher score indicates higher stress. The Cronbach’S α coefficient is 0.967, and the Split-half reliability is 0.976 [36]. The Cronbach’S α coefficients of the six dimensions are 0.842 ~ 0.925. The scale with good reliability and validity can be used to evaluate the overall stress situation of civil servants in China.

### Self-rating depression scale (SDS)

The SDS [37] is a 20-item self-report scale, which is scored on a 4-point with ranging from 1(occasionally) to 4 (constantly). Then we add up the scores of 20 items to get the total rough score and multiply the total rough score by 1.25 to get the standard score. According to the results of the Chinese norm, the abnormal threshold value of the SDS standard score is 53 points, and > 53 can be regarded as depression. The Cronbach’S α coefficient and Split-half reliability are 0.784 and 0.92, respectively [38].

### Self-rating anxiety scale (SAS)

The SAS [37] is a 20-item self-report scale, which is scored on a 4-point scale ranging from 1 (occasionally) to 4(constantly). Then we add up the scores of 20 items to get the total rough score and multiply the total rough score by 1.25 to get the standard score. According to the results of the Chinese norm, the abnormal threshold value of the SAS standard score is 50 points, and > 50 can be regarded as anxiety. Cronbach’S α coefficient is 0.767 [39].

### Connor-Davidson resilience scale (CD-RSCI)

The CD-RSCI is a 25-item self-report scale, scored on a 5-point scale ranging from 1 (never) to 5(all the time). The higher score reflects greater resilience. The Cronbach’S α coefficient of the English version is 0.89, and the coefficient of retest reliability is 0.87 [40]. The CD-RSCI scale with good reliability validity has been widely used in predicting mental resilience.

### Statistical analyses

All statistical analyses were performed using the SPSS software, version 21.0. Descriptive statistics were used to get the mean and the standard deviation. T-test and Analysis of Variance (ANOVA) were used to compare the difference in stress in the basic demographic data and the differences in stress, depression, anxiety, and resilience of civil servants at the basic level in different positions. Pearson correlations were used to explore associations among the main variables in the study. The moderating and mediating roles of resilience were analyzed in PROCESS using least squares regression [41]. To eliminate the influence of different units of data, all data were standardized before analysis.

## Results

### Demographic characteristics

Table 1 provides descriptive statistics of the detailed demographics of the participants in this study. The participants were aged between 17 and 56 years (M = 30.80, SD = 7.85) and 61.3% were male, 38.7% were female, and the sex ratio was close to 6:4. The general demographics of participants mainly included gender, age, home address, the one-child family, marital status, education, physical exercise, somatic diseases, and close relative diseases, and job characteristics, including seniority, position, relationship with coworkers, monthly income. In terms of job composition, 90.7% were clerks, and 9.3% were cadre as managers. Moreover, the difference in stress between different positions was statistically significant (*P* = 0.024); the lower position, the greater the stress. There were significant differences in gender, education, relationship with coworkers, physical exercise, and monthly income for stress (*P* < 0.05).

**Table 1:** The difference in stress in demographics

Variables		N(%)	Mean ± SD	t/F	P
Gender	Man	185 (61.3)	0.16 ± 0.89	3.414	0.001
	Female	117 (38.7)	− 0.25 ± 1.11		
Age	≤30	180 (59.6)	−0.01 ± 0.99	1.295	0.275
	31 ~ 50	115 (38.1)	0.05 ± 1.03		
	≥51	7 (2.3)	−0.57 ± 0.40		
Home	City	157 (52)	−0.01 ± 1.04	0.295	0.745
Address	Town	62 (20.5)	0.08 ± 0.70		
	Village	83 (27.5)	−0.04 ± 1.11		
The one-child	Yes	83 (27.5)	0.07 ± 0.92	0.798	0.426
	No	219 (72.5)	−0.03 ± 1.03		
Marital status	Unmarried	109 (36.1)	−0.17 ± 1.00	2.584	0.077
	Married	191 (63.2)	0.10 ± 0.99		
	Divorce	2 (0.7)	0.26 ± 0.25		
Education	Junior college	140 (46.4)	−0.24 ± 1.05	7.037	< 0.0001
	Undergraduate	152 (50.3)	0.20 ± 0.94		
	Postgraduate	10 (3.3)	0.35 ± 0.27		
Seniority	< 1	38 (12.6)	−0.30 ± 1.07	2.513	0.059
(year)	1 ~ 5	127 (42.1)	−0.01 ± 0.91		
	6 ~ 10	61 (20.2)	0.25 ± 0.99		
	> 10	76 (25.2)	−0.03 ± 1.09		
Position	Clerk	274 (90.7)	0.05 ± 0.97	2.393	0.024
	cadre	28 (9.3)	−0.54 ± 1.22		
Relationship	Good	227 (75.2)	−0.13 ± 1.01	8.053	< 0.0001
with	Average	73 (24.2)	0.37 ± 0.86		
Coworkers	Poor	2 (0.7)	0.92 ± 0.30		
Physical	Often	58 (19.2)	−0.00 ± 0.92	3.736	0.012
Exercise	By chance	173 (57.3)	0.26 ± 0.96		
	Never	71 (23.5)	0.27 ± 1.00		
Monthly	≤1500	21 (7.0)	−0.15 ± 1.25	4.238	0.006
Income (CNY)	1501 ~ 2499	128 (42.4)	−0.18 ± 1.00		
	2500 ~ 3499	75 (24.8)	0.02 ± 0.97		
	≥3500	77 (25.5)	0.32 ± 0.87		
Somatic	Yes	18 (6.0)	0.13 ± 1.03	0.559	0.577
Diseases	No	284 (94.0)	−0.01 ± 1.00		
Close relative	Yes	50 (16.6)	0.20 ± 0.94	1.567	0.118
Diseases	No	252 (83.4)	−0.04 ± 1.01		

*SD* standard deviation

### The differences in stress, depression, anxiety and resilience of grass-roots civil servants in different positions

The differences in stress, depression, anxiety, and resilience of grass-roots civil servants in different positions were shown in Table 2. There were statistically significant differences on stress (*P* = 0.024) and resilience (*P* < 0.0001). Although there was no statistical significance in depression (*P* = 0.063) and anxiety (*P* = 0.059) among civil servants of different positions, the *P* values were all close to 0.05. Table 2 suggested that the stress, depression, and anxiety in clerks were higher than that of the cadres, and the difference was statistically significant. However, the resilience of the clerks was lower than that of the cadres, and the difference was also statistically significant.

**Table 2:** A T-test on stress, depression, anxiety and resilience of civil servants at the basic level among different positions

	Position	Mean ± SD	t	P
Stress	Clerk	0.05 ± 0.97	2.393	0.024
	Cadre	−0.54 ± 1.22		
Depression	Clerk	0.03 ± 0.98	1.867	0.063
	Cadre	−0.35 ± 1.13		
Anxiety	Clerk	0.03 ± 1.01	1.898	0.059
	Cadre	−0.35 ± 0.80		
Resilience	Clerk	−0.06 ± 0.97	−3.697	< 0.0001
	Cadre	0.68 ± 1.09		

*SD* standard deviation

### The correlation between stress, anxiety, depression and resilience

The scores of SDS ranged from 30 to 89 that the mean is 54.47 (SD = 9.84), and the scores of SAS ranged from 25 to 91, that the mean is 48.23 (SD = 11.17). One hundred ninety-seven civil servants (65.2%) had depression, the standard score of SDS > 53, and 127 civil servants (42.1%) had anxiety, the standard score of SAS > 50. Pearson correlation analysis revealed (Table 3) that stress was positively correlated with depression and anxiety (*P* < 0.001). Conversely, stress was negatively correlated with resilience (*P* < 0.001). Resilience was negatively correlated with depression and anxiety (*P* < 0.001).

**Table 3:** Pearson correlations coefficients of stress, depression, anxiety and depression among grass-roots civil servants(r)

Variables	Mean ± SD	1	2	3
1.stress	149.52 ± 65.89			
2.depression	0.54 ± 0.10	0.212***		
3.anxiety	48.23 ± 11.17	0.444***	0.699***	
4.resilience	48.06 ± 9.32	−0.212***	−0.382***	− 0.343***

*SD* standard deviation

Note: *P < 0.05, **P < 0.01, ***P < 0.001

### Mediating effects of resilience in the relationship between stress and depression, anxiety

As shown in Table 4, two mediation analyses were performed. Depression (as the dependent variable), home address, degree of education, physical exercise, monthly income, and illness (as covariates), stress (as an independent variable), and resilience (as mediator) were entered into model 59. The results indicated that resilience played a partial mediating role in the relationship between stress and depression and the direct effect was 77.21%, and the indirect effect was 22.79%. Secondly, anxiety (dependent variable), relationship with colleagues, physical exercise, monthly income (covariates), stress (independent variable), and resilience (mediator) were entered. The resilience had shown mediation between stress and anxiety, and the direct effect was 92.23%, and the indirect effect was 7.77%.

**Table 4:** Mediating effects of resilience in the relationship between stress, depression, and anxiety

Effect	Depression			Anxiety		
	β (SE)	*P*	95%CI	β (SE)	*P*	95%CI
a	−0.22 (0.57)	<0.001		−0.19 (0.06)	0.001	
b	−0.30 (0.05)	<0.001		−0.21 (0.05)	<0.001	
C′	0.22 (0.05)	<0.001		0.46 (0.05)	<0.001	
a*b	0.06 (0.02)		(0.020,0.115)	0.04 (0.02)		(0.009,0.078)

Note: * inside Table 4 means the effects of resilience in the relationship between stress and depression and anxiety

### Moderating effects of resilience in the relationship between stress, depression, and anxiety

As presented in Table 5, two moderation analyses were performed. Depression (dependent variable), home address, degree of education, physical exercise, monthly income, and illness (covariates), stress (independent variable), and resilience (moderator) were entered into model 59. The interaction effect was significant in stress and resilience on depression (*P* < 0.001). Secondly, anxiety (dependent variable) relationship with colleagues, physical exercise, and monthly income (covariates), stress (independent variable), and resilience (moderator) were entered. The interaction effect was significant in stress and resilience on anxiety (*P* < 0.001). Thus, the hypothesis that resilience moderates between stress, depression, and anxiety are valid.

**Table 5:** Moderating effects of resilience in the relationship between stress, depression, and anxiety

Predictors	Depression				Anxiety			
	β (SE)	*P*	*R*^*2*^	*F*	β (SE)	*P*	*R*^*2*^	*F*
Stress	0.23 (0.05)	<0.001	0.280	14.271	0.47 (0.05)	<0.001	0.358	23.396
Resilience	−0.34 (0.05)	<0.001			−0.25 (0.05)	<0.001		
Stress×Resilience	−0.13 (0.04)	0.001			−0.13 (0.04)	<0.001		

**Fig. 2:**
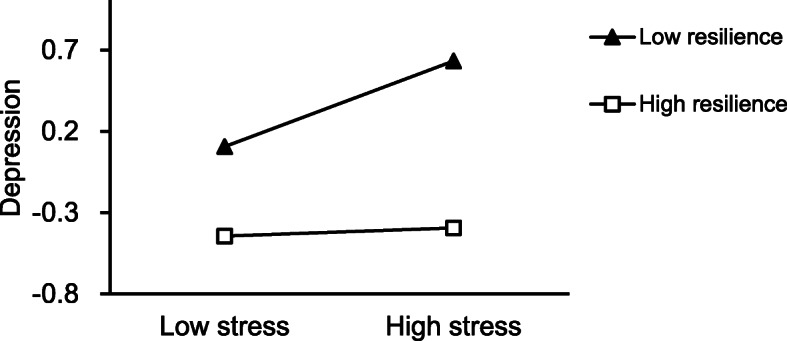
The relationship between stress and depression across different resilience group

Two hierarchical linear regression models were performed to analyze further the moderating effect of resilience on the relationship between depression and anxiety. Stress and resilience were classified as high (M + SD) and low (M-SD). Firstly, the dependent variable was depression, as shown in Fig. 2. The estimates of 95% bias-corrected bootstrap CI of M-SD and M + SD were (0.2231, 0.4936) and (− 0.0337, 0.2219), respectively. The difference of 95% bias-corrected bootstrap CI of M-SD and M + SD was (0.2568, 0.2712). There was no zero in the difference of 95% bias-corrected bootstrap CI, which indicated that resilience had made a moderation effect between stress and depression. Moreover, stratified analysis results of resilience showed that grass-roots civil servants with lower resilience were more susceptible to stress, and higher stress was more likely to make them depressed.

**Fig. 3:**
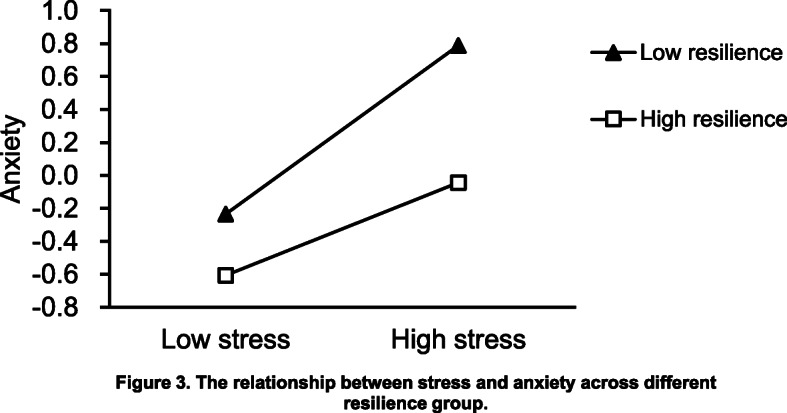
The relationship between stress and anxiety across different resilience group

Secondly, the dependent variable was anxiety, as shown in Fig. 3. The estimates of 95% bias-corrected bootstrap CI of M-SD and M + SD were (0.4754, 0.7337) and (0.2219, 0.4613), respectively. The difference of 95% bias-corrected bootstrap CI of M-SD and M + SD was (0.2535, 0.2724). There was no zero in the difference of 95% bias-corrected bootstrap CI, which indicated that resilience had made a moderation effect between stress and anxiety. The stratified analysis results of resilience showed that the increase of stress led to anxiety of low or high elasticity. However, the increase of stress that led to anxiety in the low resilience group was higher than in the high resilience group.

## Discussion and limitations

Our study found that there were 65.5 and 42.1% of grass-roots civil servants indicating depression and anxiety, respectively. The results are consistent with previous findings in China that civil servants generally have high levels of depression and anxiety [6]. Meanwhile, although the research focus on civil servants in different countries is different, it is found that civil servants suffer from depression, anxiety, and other psychological problems commonly [42–44]. However, this phenomenon has not received much attention, especially the grass-roots civil servants of this special group. So, the primary purpose of the study was to explore the psychosocial correlative factor of the stress in grass-roots civil servants, especially the resilience and its influence on the relationship between stress and anxiety and depression among grass-roots civil servants.

There were statistically significant differences in gender, education, position, relationship with coworkers, physical exercise, and monthly income among the stress of the grass-roots civil servants. Among the grass-roots civil servants, 61.3% were men, 38.7% were women, 90.7% were clerks, 9.3% were cadres. Men were more stressed than women, and the difference was statistically significant, which was in agreement with previous researches [45, 46]. In China, the income of men is one of the main sources of family income generally, and they tend to feel more stressed than women. Hence, the stress of men of grass-roots civil servants is generally higher than women. Also, most grass-roots civil servants were clerks, and cadres were only a small part of them. The high education level of civil servants at the basic level has certain advantages in promoting and social welfare, while those with lower education have relatively high professional competitive pressure. Excluding those with outstanding ability and higher education, most clerks are less likely to be promoted, and most of them take a basic salary to support their families. Compared with cadres, the general staff have not only the pressure of work and the pressure of promotion. Therefore, the stress of grass-roots civil servants in different positions was different, and the difference was statically significant, and the stress of clerks was higher than that of cadres.

The *P*-value of depression and anxiety of different positions of the grass-roots civil servants were close to 0.05. That may be due to the relatively small sample size. The P-value was approaching to or even less than 0.05 when the sample size increased. The differences in stress, depression, anxiety, and resilience of the grass-roots civil servants in different positions showed that clerks felt more stress, depression, and anxiety than cadres, and their resilience was relatively low. Moreover, long-term relatively high stress, depression, and anxiety will not only reduce work efficiency but also affect physical health [47, 48]. The mental health status of clerks in the grass-roots civil servants is relatively poor and their resilience needs to be improved.

As shown in Fig. 1, the stress of the grass-roots civil servants is positively correlated with depression, and anxiety, while resilience is negatively corrected with stress, depression and anxiety, which indicates that stress in the grass-roots civil servants is a risk factor for depression and anxiety, while resilience is a protective factor for depression and anxiety. This is consistent with previous studies of depression and anxiety [4, 5, 19, 32]. Stress is a risk factor for depression and anxiety. To some extent, stress can cause and aggravate depression and anxiety. It can also be said that the grass-roots civil servants with depression and anxiety, to a certain extent, have relatively low ability to face and negotiate pressure to overcome adverse consequences. That is to say, the hypothesis that resilience moderated the relationship between stress and depression and anxiety has been confirmed. Previous studies have also found that resilience can partially moderate stress and other psychological and emotional problems [6, 28]. To some extent, resilience can relieve depression and anxiety caused or aggravated by stress. The grass-roots civil servants with high resilience may have more ability to deal with the stress in life and work than those with low resilience, thereby reducing the risk of depression and anxiety.

No study has investigated the impact of resilience on the relationship between the stress, depression, and anxiety of grass-roots civil servants. Some research have shown that resilience played a partial mediating role in the relationship between stress and psychological health [49, 50]. However, in addition to the mediating role, there may be other types of roles of resilience in the relationship between stress and depression and anxiety, including direct effect and moderate effect. Therefore, this study not only used a statistical model to explore the role of resilience but also adopted a hierarchical linear regression model to further test the moderating effect of resilience. The mediation analysis found that resilience had more than three times an effect on depression than anxiety, suggesting that resilience was more important than anxiety in explaining depression. The hierarchical linear regression model showed that when the effect of stress on the depression of the grass-roots civil servants with higher, resilience scores were lower, while those with lower resilience scores were significantly higher in the relationship between stress and depression. The grass-roots civil servants in the high resilience group scores were lower on the relationship between stress and anxiety than those in the low resilience group. Through moderating and mediating analysis, it is found that resilience can not only directly affect the relationship between stress, depression, and anxiety, but also can indirectly affect the relationship too. Resilience can directly block the negative impact of stress on mental health and thus become a protective factor of mental health under stress. The relationship between stress, depression, and anxiety supports compensatory and protective models of resilience. This is also consistent with the results of previous resilience model studies that the higher score of resilience, better able to resist the negative effect of stress, and the more positive the psychological health [6, 28, 51]. However, in the study of resilience in adolescents, resilience only regulating the relationship between stress and depression not between stress and anxiety [32]. It may be due to different participants and different countries and other factors. The reason why resilience can be an intrinsic protective factor is possible that individuals with higher resilience are better at assessing stressful events in a positive cognitive way in the face of the same stress. They tend to adjust the relationship between the environment and individuals more actively, stimulate and promote their potential, make full use of various resources, face pressure, and achieve a good state of adaptation and development. As a result, resilience is one of the factors that could be considered in intervention programs to improve psychological health in grass-roots civil servants in the future. Enhancing individual resilience level, improving education level, maintaining a good relationship with colleagues, increasing physical exercise, and increasing monthly income can reduce the pressure of grass-roots civil servants from the source and prevent the occurrence of mental diseases.

This study has some limitations. First, it is a cross-sectional study, which means that causality cannot be determined. Second, because of the self-reporting questionnaire used in the current study, the results may be constrained by memory bias and individual subjective consciousness. Finally, the sample size of this article is too small, with only one county, to represent the grass-roots civil servants all over the country. The next step is to make a cohort-study and expand the investigation scope to increase the sample representation. Future studies can explore mechanismS and processes of resilience and can look for ways to enhance the resilience of grass-roots civil servants.

## Conclusion

In conclusion, through the analysis of the resilience of grass-roots civil servants in the context of stress and its influence on anxiety and depression, the results showed that resilience played the moderating and mediating roles in the relationship between stress and depression and anxiety. The negative effects of stress on depression and anxiety of grass-roots civil servants could be buffered by resilience, directly and indirectly, which is a dynamic moderate mode. Furthermore, resilience can be bolstered and targeted for prevention efforts. Improving resilience and reducing stress plays a vital role in preventing depression and anxiety in the grass-roots civil servants. These findings can help the society and government departments better understand the mental health status of grass-roots civil servants and provide references and guidance for the formulation of corresponding management and prevention measures, and create a high-level working environment for grass-roots civil servants.

## Data Availability

The datasets used and /or analyzed during the current study are available from the corresponding author on reasonable request.

## Abbreviations

CSSS: Civil Servants Stress Scale
SDS: Self-rating Depression Scale
SAS: Self-rating Anxiety Scale
CD-RISC: Connor-Davidson Resilience Scale
CNY: Chinese yuan
